# Mycotoxins and the Intestinal Epithelium: From Barrier Injury to Stem Cell Dysfunction

**DOI:** 10.3390/toxins17110534

**Published:** 2025-10-30

**Authors:** Wenying Huo, Yingying Qiao, Xiangru He, Cailing Wang, Ruiqing Li, Long Che, Enkai Li

**Affiliations:** 1 College of Animal Science and Technology, Henan University of Animal Husbandry and Economy, Zhengzhou 450046, China; q18236799337@163.com (Y.Q.); cailing@188.com (C.W.); liruiqing2003@163.com (R.L.); chelong1989@126.com (L.C.); 2 Department of Statistics, Graduate School of Arts and Sciences, Columbia University, New York, NY 10032, USA; xh2708@columbia.edu; 3 Department of Molecular Microbiology, Washington University School of Medicine, St. Louis, MO 63110, USA

**Keywords:** mycotoxins, intestinal barrier, oxidative stress, inflammation, intestinal stem cells

## Abstract

Mycotoxins are toxic secondary metabolites produced by filamentous fungi that contaminate agricultural commodities, posing risks to food safety, animal productivity, and human health. The gastrointestinal tract is the first and most critical site of exposure, where the intestinal epithelium functions as both a physical and immunological barrier against luminal toxins and pathogens. While extensive research has demonstrated that mycotoxins disrupt epithelial integrity through tight junction impairment, oxidative stress, apoptosis, and inflammation, their effects on the intestinal stem cell (ISC) compartment and epithelial regeneration remain insufficiently understood. This review integrates recent findings from in vivo, cell culture, and advanced 3D intestinal organoid and gut-on-chip models to elucidate how mycotoxins such as deoxynivalenol and zearalenone impair ISC proliferation, alter Wnt/Notch signaling, and compromise mucosal repair. We also discuss dose relevance, species differences, and the modulatory roles of the microbiome and short-chain fatty acids, as well as emerging evidence of additive or synergistic toxicity under co-exposure conditions. By bridging well-established mechanisms of barrier disruption with the emerging concept of ISC-driven regenerative failure, this review identifies a critical knowledge gap in mycotoxin toxicology and highlights the need for integrative models that link epithelial damage to impaired regeneration. Collectively, these insights advance understanding of mycotoxin-induced intestinal dysfunction and provide a foundation for developing nutritional, microbial, and pharmacological strategies to preserve gut integrity and repair.

## 1. Introduction

Mycotoxins are secondary metabolites of fungi that contaminate a wide range of food and feed commodities, with surveys repeatedly detecting them in cereals, animal diets, and derived products across continents [1,2,3]. Their global prevalence poses a dual challenge: economic losses in the feed industry due to reduced animal performance, and serious health concerns for both livestock and humans [2,4]. In farm animals, chronic exposure is associated with impaired growth, reproductive disorders, and increased disease susceptibility, while in humans, dietary intake has been linked to gastrointestinal dysfunction, hepatotoxicity, immunosuppression, and even carcinogenesis depending on dose, toxin type, and age at exposure [3,4]. The frequent co-occurrence of multiple mycotoxins further complicates risk assessment, as combined exposures may produce additive or synergistic toxic effects [1,4].

Among target organs, the intestine is particularly vulnerable because of its direct contact with ingested toxins. Experimental and in vivo studies consistently demonstrate that trichothecenes such as deoxynivalenol (DON) and nivalenol disrupt epithelial integrity by downregulating tight junction proteins and increasing paracellular permeability, ultimately compromising the mucosal barrier [5,6]. These toxins also stimulate excessive production of reactive oxygen species (ROS) in intestinal epithelial cells, producing oxidative stress that damages cellular components and amplifies barrier dysfunction [7,8]. Oxidative stress and epithelial injury converge on proinflammatory signaling cascades, including NF-κB activation and cytokine release, which further impair gut function [6,7]. Importantly, early-life or maternal exposure can exacerbate these effects: maternal zearalenone (ZEN) exposure in rodents has been shown to induce villus atrophy, crypt remodeling, immune dysregulation, and long-term morphological alterations in offspring intestines, suggesting that developmental exposure generates persistent vulnerabilities [4,8].

In parallel with toxicological research, significant progress has been made in understanding the biology of intestinal stem cells (ISCs), which underpin epithelial homeostasis and regeneration. Actively cycling Lgr5+ crypt base columnar cells provide the primary source of continuous epithelial renewal, while quiescent “reserve” stem cells can be mobilized under conditions of severe injury [9,10]. ISC activity is orchestrated by niche-derived signals, including Wnt, Notch, and EGF from Paneth cells and stromal elements, as well as modulatory influences from immune cells and the microbiota [9,10]. Following injury, such as radiation exposure, ISCs mount a rapid regenerative response to restore the epithelium, underscoring their essential role in maintaining intestinal integrity [10,11].

Despite these advances, the intersection between mycotoxin toxicology and ISC biology remains underexplored. While numerous studies have documented the ability of DON, ZEN, and other mycotoxins to induce oxidative stress, inflammation, and barrier dysfunction in differentiated epithelial cells, little is known about their direct effects on ISCs, including their survival, proliferative dynamics, lineage differentiation, and responsiveness to niche signals [5,6,9]. Given that the regenerative capacity of the gut depends on ISC function, elucidating how mycotoxins affect these stem cell populations represents a critical knowledge gap. Addressing this gap is essential to understanding the long-term impact of chronic or developmental mycotoxin exposure on intestinal health and developing strategies that mitigate these risks in both humans and animals. This review synthesizes current knowledge on the effects of mycotoxins on the intestinal epithelium, progressing from well-characterized barrier disruption to the less explored but highly consequential impairment of ISC-driven regeneration. By integrating insights from in vivo studies, cell lines, and organoid models, we highlight emerging mechanisms, research gaps, and implications for both human and animal health.

## 2. Cell Type-Specific Mechanisms of Mycotoxin Toxicity in the Intestine

### 2.1. Mycotoxin-Induced Alterations in Intestinal Epithelial Cells

Deoxynivalenol (DON) and other mycotoxins exert profound effects on multiple intestinal cell populations (Table 1), disrupting both barrier function and regenerative capacity. In enterocytes, DON inhibits protein synthesis, leading to impaired tight junction assembly and increased epithelial permeability, while simultaneously inducing endocytosis and lysosomal degradation of junctional proteins through selective activation of MAPK signaling pathways, thereby compromising barrier integrity and epithelial homeostasis [12,13,14]. Zearalenone (ZEA) further perturbs enterocyte proliferation and differentiation by modulating Wnt/β-catenin signaling, indicating that both trichothecenes and estrogenic mycotoxins can impair epithelial renewal [15]. Goblet cells are highly sensitive to DON, which suppresses mucin and trefoil factor (TFF) expression via PKR- and MAP kinase-dependent pathways, weakening the mucus layer and reducing the protective barrier against microbial invasion [16,17,18]. Paneth cells serve a critical protective role for the ISC niche; they mitigate DON-induced oxidative stress and preserve ISC viability, thereby maintaining the regenerative capacity of the crypt [19]. Enteroendocrine cells respond to DON exposure by releasing gut hormones such as peptide YY, cholecystokinin, and glucagon-like peptide-1 through calcium-sensing receptor and transient receptor potential ankyrin-1 channel-mediated pathways, which modulate satiety signaling and gut-brain communication [20,21]. ISCs themselves are direct targets of mycotoxins: DON restricts ISC proliferation and regenerative potential via IP3R-dependent Ca^2+^ signaling in the endoplasmic reticulum and through TSC2/mTORC1 pathways activated by insulin and EGFR receptors, highlighting both intracellular and receptor-mediated mechanisms [22,23]. Together, these findings demonstrate that mycotoxins disrupt multiple intestinal cell types—including enterocytes, goblet cells, Paneth cells, enteroendocrine cells, and ISCs—compromising epithelial barrier integrity, mucus secretion, hormonal signaling, and regenerative capacity, with Paneth cell-mediated niche support emerging as a key protective factor against mycotoxin-induced intestinal injury [19,24].

### 2.2. Mycotoxin-Induced Alterations in Intestinal Intraepithelial Lymphocytes (IELs) and Immune Signaling

Mycotoxins exert diverse and often immunosuppressive effects on intestinal intraepithelial lymphocytes (IELs) and mucosal cytokine responses, compromising epithelial immune surveillance (Table 2). In broilers, diets co-contaminated with deoxynivalenol (DON) and zearalenone (ZEA) significantly reduced the proportion of CD3+, CD4+, and CD8+ T cells in the duodenal epithelium, indicating impaired local adaptive immunity and disruption of IEL-mediated barrier defense [25]. Similarly, feeding chickens Fusarium-contaminated diets suppressed intestinal CD4+ helper and CD8+ cytotoxic T-cell subsets and reduced lymphocyte proliferation, alongside atrophy of lymphoid organs such as the bursa and thymus, pointing to a systemic impact of these toxins on cell-mediated immunity [26]. In swine, even a low oral dose of T-2 toxin (15 µg/kg b.w./day) altered the relative proportions of T and B lymphocytes in the ileal wall and modulated cytokine release, with reduced IL-2 and IFN-γ alongside enhanced IL-4 expression, suggesting a toxin-driven shift from Th1 toward Th2 responses [27]. Ochratoxin A (OTA) induced marked lymphoid depletion in mucosa-associated tissues of chickens, including Peyer’s patches and cecal tonsils, accompanied by decreased CD3+ IELs and histopathological evidence of villus atrophy, crypt hyperplasia, and mucosal necrosis, collectively weakening gut-associated lymphoid tissue (GALT) function [28]. Aflatoxin B1 (AFB1) exerted potent immunotoxicity in broilers by downregulating intestinal CD3+, CD4+, and CD8+ T-cell subsets and disrupting cytokine transcription, with suppressed IL-2 and IFN-γ but elevated IL-6, reflecting both impaired T-cell signaling and enhanced proinflammatory stress [29]. In murine models, DON showed a biphasic effect, stimulating lymphocyte activation and cytokine production at subtoxic levels but causing T-cell apoptosis, decreased proliferation, and suppression of Th1 cytokines at higher doses [30]. ZEA exposure in mice further altered IEL homeostasis by modulating estrogen receptor-mediated signaling, skewing T-helper responses, and disrupting the CD4+/CD8+ balance while altering cytokine expression profiles [31]. Across studies, these data collectively underscore that mycotoxins target both IEL subsets and mucosal cytokine networks through distinct but overlapping mechanisms—including oxidative stress, receptor-mediated signaling, and direct cytotoxicity—resulting in weakened intestinal barrier immunity and heightened vulnerability to enteric infections.

## 3. Mycotoxin-Induced Intestinal Barrier Injury

Mycotoxins, including DON, ZEA, T-2, HT-2, aflatoxin B1, aflatoxin M1, and ochratoxin A, profoundly impair intestinal barrier integrity, primarily through disruption of tight junction (TJ) proteins such as claudins, occludin, and ZO-1 (Table 3). DON inhibits protein synthesis and triggers endocytosis and degradation of TJ proteins via activation of MAPK pathways, mitochondrial dysfunction, and hijacking of PGC1α-mediated signaling, leading to increased paracellular permeability and barrier leakage [12,34,35]. Zearalenone acutely disturbs barrier homeostasis by modulating the Wnt/β-catenin signaling pathway, while T-2 and HT-2 toxins similarly compromise epithelial integrity through direct cytotoxicity [15,36]. Aflatoxins reduce expression of TJ-related genes and induce apoptosis, whereas ochratoxin A selectively removes claudin isoforms, further weakening the barrier [37,38,39,40]. Beyond structural disruption, DON affects specialized epithelial cells, repressing mucin production and trefoil factor (TFF) expression in goblet cells through PKR- and MAPK-dependent pathways, and altering Paneth cell function, thereby exacerbating vulnerability to luminal toxins and microbes [16,17,19,41]. Nutritional and pharmacological interventions demonstrate protective effects: quercetin, kaempferol, EPA, DHA, and PPARγ activation stabilize TJs by inhibiting claudin endocytosis and activating Nrf2/PPARγ pathways, while galacto-oligosaccharides maintain TJ networks and suppress inflammatory responses. Mycotoxin binders enhance TJ gene expression in vivo, collectively mitigating barrier dysfunction [42,43,44,45,46,47,48]. These findings underscore that mycotoxins disrupt intestinal integrity through multiple complementary mechanisms and highlight the potential of targeted nutritional, microbial, and pharmacological strategies to protect or restore barrier function.

## 4. ISCs: Mycotoxin Impact and Nutritional Protection

### 4.1. ISCs in Homeostasis and Response to Mycotoxin

ISCs reside at the base of the intestinal crypts, where actively cycling Lgr5+ crypt base columnar cells serve as the main drivers of continuous epithelial renewal, supported by a quiescent population of reserve stem cells that can be mobilized under stress or severe injury [9,10]. Lgr5+ ISCs divide to produce transit-amplifying progenitors that undergo tightly regulated proliferation and differentiation, generating all major epithelial lineages, including absorptive enterocytes, mucus-secreting goblet cells, antimicrobial Paneth cells, and hormone-producing enteroendocrine cells, ensuring maintenance of epithelial structure and function [9]. ISC activity is tightly controlled by a specialized niche: Paneth cells provide essential Wnt, Notch, and EGF signals, while stromal cells, immune cells, and microbiota-derived factors modulate stem cell proliferation, differentiation, and responsiveness to environmental cues, highlighting the complexity of ISC regulation in homeostasis [9,10]. ISCs are critical for regenerative responses following injury or insult: radiation activates both Lgr5+ active and reserve ISCs to repopulate damaged epithelium [10,11]. However, they are vulnerable to environmental and microbial toxins; Clostridioides difficile toxin B directly impairs colonic stem cells, hindering repair [50], enterotoxigenic E. coli heat-stable enterotoxin inhibits ISC expansion by downregulating Wnt/β-catenin signaling [51], and mycotoxins such as deoxynivalenol (DON) induce oxidative stress that disrupts ISC function and compromises epithelial regeneration [52]. Together, these findings highlight ISCs as central regulators of intestinal homeostasis and repair, yet also as direct targets of toxins and pathogens, emphasizing the need to understand mechanisms governing their resilience and susceptibility.

### 4.2. Nutritional Interventions Enhancing ISC Function During Mycotoxin Exposure

Nutritional interventions have been demonstrated to effectively counteract deoxynivalenol (DON)-induced ISC injury by restoring proliferation, differentiation, and regenerative potential through multiple molecular mechanisms (Table 4). Recombinant porcine R-Spondin 1 promotes ISC expansion along the crypt–villus axis by potentiating Wnt/β-catenin signaling, enhancing the expression of target genes involved in stem cell self-renewal, and restoring crypt architecture under both homeostatic and DON-challenged conditions [53]. Butyrate, a short-chain fatty acid, mitigates DON-induced epithelial barrier dysfunction in jejunal organoid-derived monolayers by supporting ISC survival, indirectly reinforcing epithelial regeneration, and maintaining barrier integrity [54,55,56]. Hydrolyzed wheat gluten and zinc L-aspartate activate Wnt/β-catenin signaling, leading to increased ISC proliferation and differentiation, thereby preserving crypt function and mucosal integrity during mycotoxin exposure [57,58,59,60,61]. Lauric acid alleviates DON-induced ISC damage through potentiation of the Akt/mTORC1/S6K1 signaling axis, which promotes protein synthesis and cell growth essential for stem cell maintenance and epithelial repair [62]. Methionine and its hydroxyl analogues similarly reactivate Wnt/β-catenin signaling, improving ISC activity and enabling recovery from DON-induced injury by restoring proliferative capacity and crypt–villus dynamics [57]. Furthermore, L-carnosine protects ISCs from oxidative stress by regulating the Keap1/Nrf2 signaling pathway, reducing reactive oxygen species accumulation, and maintaining stem cell viability and function [63,64,65]. Collectively, these findings underscore that targeted nutritional strategies—ranging from growth factor supplementation, amino acids, fatty acids, minerals, to antioxidants—can effectively mitigate DON-induced ISC dysfunction, primarily by enhancing Wnt/β-catenin and Akt/mTORC1 signaling pathways and reducing oxidative stress, thereby sustaining intestinal regeneration, barrier function, and epithelial homeostasis.

## 5. Advantages of Organoids and Dynamic 3D Systems over Conventional 2D Cultures in Modeling Mycotoxin Toxicity

Conventional 2D intestinal monolayers have provided foundational insights into mycotoxin toxicity, particularly for barrier disruption, but they fall short in replicating the complex architecture, cellular heterogeneity, and dynamic environment of the gut epithelium. As a result, their ability to capture subtle effects on specialized epithelial cell populations and ISCs is limited. Recent studies underscore the advantages of 3D organoid and microphysiological systems, which more closely mimic the crypt–villus structure, multicellular composition, and luminal exposure relevant to in vivo conditions (Table 5). Reviews emphasize that organoids preserve critical signaling pathways such as Wnt, Notch, and EGF that regulate ISC renewal, making them superior for evaluating toxin-driven epithelial injury [66]. Direct comparative studies demonstrate that 3D spheroids exhibit greater sensitivity to sterigmatocystin, ochratoxin A, and patulin than 2D monolayers, revealing stronger cytotoxic responses and more physiologically relevant dose–response profiles [65,67,68,69].

Species-specific organoid platforms have been instrumental in broadening toxicological relevance beyond human models. Bovine apical-out organoids revealed luminal deoxynivalenol (DON) toxicity and provided a system for testing probiotic detoxification strategies [70], while porcine organoid-derived monolayers demonstrated that DON and T-2 facilitate bacterial translocation across the epithelium, highlighting complex pathogen–toxin interactions [71]. Sheep intestinal organoids were recently developed as the first ovine system to study DON toxicity [72], and chicken intestinal organoids successfully recapitulated DON-induced barrier disruption in poultry [73], offering new tools for livestock-specific assessments. Importantly, organoid-based systems have also enabled evaluation of dietary interventions, as shown by butyrate supplementation, which alleviated DON-induced epithelial barrier dysfunction in pig jejunum organoid monolayers [54].

Beyond static culture, gut-on-a-chip and microfluidic systems integrate flow, dose dynamics, and mechanical stimulation, thereby simulating in vivo toxin exposure with higher fidelity. DON exposure in gut-on-chip models revealed dose- and route-dependent toxicity that could not be reproduced in flat cultures [74], while microfluidic systems provided continuous toxin perfusion, enabling fine-scale analysis of barrier injury and cytotoxicity [67,75,76]. Collectively, these advances establish 3D organoid and gut-on-chip models as indispensable tools for dissecting how mycotoxins compromise gut barrier function and ISC-driven regeneration, while also providing platforms to evaluate nutritional and microbial mitigation strategies.

**Table 5:** Advantages of organoid and advanced 3D systems over 2D monolayers for studying mycotoxin-induced gut barrier dysfunction and intestinal stem cell injury.

Model Type	Species	Mycotoxin(s)	Key Insight	Reference
3D organoids & microphysiological systems	Human/Animal	Multiple	3D systems replicate epithelial complexity and the ISC niche, providing more relevant toxicity outcomes.	[66]
3D spheroids vs. 2D monolayers	Human	Sterigmatocystin, Ochratoxin A, Patulin	3D spheroids show higher sensitivity and more physiologically relevant cytotoxic responses than 2D.	[75]
Apical-out intestinal organoids	Bovine	DON	Captures luminal DON exposure and supports probiotic detoxification, unlike standard 2D.	[70]
Organoid-derived monolayers	Porcine	DON, T-2	Reveals DON/T-2-driven bacterial translocation, difficult to replicate in 2D.	[71]
Organoid-derived cell monolayers	Pig jejunum	DON	Butyrate supplementation mitigates DON-induced barrier dysfunction more effectively in organoid-derived models.	[54]
3D spheroids	Human (tumor & healthy)	Sterigmatocystin, Ochratoxin A, Patulin	Co-exposure to multiple mycotoxins induces stronger cytotoxicity in 3D vs. 2D.	[67]
Microfluidic 3D culture system	Human	Ochratoxin A, Patulin	Microfluidics allows dynamic exposure mimicking physiological flow, outperforming static 2D assays.	[68]
Intestinal organoids	Sheep	DON	First ovine organoid system showing DON toxicity, advancing livestock-specific toxicology.	[72]
Intestinal organoids	Chicken	DON	Captures DON-induced barrier dysfunction in poultry, not feasible with simple 2D.	[73]
Gut-on-a-chip with flow	Human	DON	Demonstrates dose- and route-dependent DON toxicity under flow, beyond static 2D limitations.	[74]
High-throughput gut-on-chip	Human	Enterotoxins (applicable to mycotoxins)	High-throughput, physiologically dynamic toxin assessment superior to flat monolayers.	[76]

## 6. Mycotoxins and Intestinal Health: Physiological Relevance, Co-Exposure, and Regeneration

### 6.1. Physiological Relevance of Experimental Concentrations

Mycotoxin concentrations applied in vitro (typically 0.1–10 µM for DON, ZEA, and OTA; 0.01–1 µM for T-2 toxin; and up to 5 µM for AFB1) often exceed expected intestinal luminal levels following normal dietary exposure but are necessary to reproduce acute or subchronic epithelial injury [5,41]. In vivo, dietary inclusion levels of 0.5–5 mg/kg for DON or 0.1–1 mg/kg for ZEA reliably induce intestinal and immunological effects in pigs and poultry, consistent with their high sensitivity compared to ruminants [15]. Human dietary exposure is generally several orders of magnitude lower, with mean estimated daily intakes for DON (0.01–0.1 µg/kg body weight) well below the tolerable daily intake (1 µg/kg body weight/day, EFSA). These comparisons emphasize that while mechanistic studies use supraphysiological doses to elicit measurable effects, they provide valuable insight into pathways that may become relevant under chronic, co-exposure, or stress-amplified conditions (Table 6).

### 6.2. Mycotoxin Co-Exposure and Combined Toxicity

In real-world settings, animals and humans are rarely exposed to a single mycotoxin. Co-occurrence of trichothecenes (e.g., DON, T-2), zearalenone (ZEA), fumonisins, and aflatoxins in feed and food is common, leading to additive or synergistic effects on intestinal integrity and immune function. Experimental data indicate that combined DON and ZEA exposure amplifies epithelial injury by intensifying oxidative stress, proinflammatory cytokine release, and tight junction disruption beyond single-toxin effects [77,78,79]. Similarly, mixtures of DON, ZEA, and fumonisin B1 in poultry and pigs exert synergistic suppression of intraepithelial lymphocytes and greater barrier dysfunction, highlighting cross-talk between shared signaling pathways such as MAPK, NF-κB, and Wnt/β-catenin [41,58]. Conversely, some interactions may be antagonistic under subtoxic conditions due to metabolic competition for detoxification enzymes [6]. Collectively, co-exposure studies reveal that the intestinal impact of mixed mycotoxins cannot be predicted from individual toxicities alone, underscoring the importance of integrated risk assessment using physiologically relevant mixtures in organoid or gut-on-chip systems.

### 6.3. Microbiome- and SCFA-Mediated Modulation of Epithelial Barrier and Intestinal Stem Cells

The gut microbiome profoundly influences epithelial resilience to mycotoxins through metabolic and signaling interactions. Commensal bacteria degrade certain mycotoxins—such as DON and ZEA—into less toxic derivatives, while dysbiosis induced by chronic exposure can reduce this detoxification capacity [54,57]. Microbial metabolites, especially short-chain fatty acids (SCFAs) like butyrate, acetate, and propionate, play dual protective roles: they reinforce tight junction integrity by activating AMPK and Nrf2-dependent antioxidant pathways and promote ISC proliferation and differentiation through Wnt/β-catenin and Notch signaling modulation [19,80,81]. Butyrate, in particular, has been shown in pig jejunum organoid-derived monolayers to ameliorate DON-induced epithelial barrier dysfunction by preserving ISC survival and enhancing crypt regeneration [54]. These findings highlight a dynamic interplay between microbial metabolism, epithelial regeneration, and toxin susceptibility, suggesting that microbiota-targeted interventions could mitigate mycotoxin-induced intestinal injury.

Despite advances in understanding acute mycotoxin-induced epithelial damage, significant gaps remain regarding long-term regenerative dysfunction. Most studies have focused on DON, while other mycotoxins—including fumonisins, zearalenone, T-2 toxin, ochratoxin A, and emerging compounds such as enniatins and beauvericin—remain largely unexplored in the context of ISC impairment [52]. Key limitations include species-specific differences, a lack of longitudinal studies, incomplete mechanistic insights, and limited consideration of microbiota and dietary interactions [82].

Intestinal stem cell markers, such as Lgr5, Olfm4, and Bmi1, hold promise as early biomarkers of mycotoxin-induced toxicity, as alterations in these markers may precede overt epithelial damage and provide mechanistic insight into compromised regenerative capacity [83]. Dose- and time-dependent studies indicate that low to moderate toxin exposures can selectively impair ISC proliferation and niche function, while higher concentrations or chronic exposures cause generalized epithelial injury, including apoptosis, barrier disruption, and inflammation [19]. Acute exposures typically trigger transient ISC dysfunction and reversible epithelial damage, allowing for rapid regeneration once the toxin is removed. Chronic or repeated exposures, however, lead to persistent ISC suppression, niche disruption, and cumulative damage, slowing or preventing full epithelial recovery. The interplay between epithelial injury and impaired ISC renewal is critical: ongoing epithelial loss without adequate stem cell-mediated repair prolongs barrier defects and promotes inflammation, which in turn further suppresses ISC function, creating a vicious cycle that impedes long-term gut recovery.

Dietary and bioactive interventions can support ISC-driven regeneration under mycotoxin exposure. Lauric acid alleviates DON-induced ISC injury by potentiating the Akt/mTORC1/S6K1 signaling axis, while methionine and its analogues reactivate Wnt/β-catenin signaling, enhancing ISC activity and recovery [22]. Other acids, including butyrate, caprylic and capric acids, glutamic acid, alpha-ketoglutarate, and omega-3 fatty acids, provide energy, antioxidant, and anti-inflammatory support to preserve ISC function. Probiotics (e.g., Lactobacillus, Bifidobacterium, Bacillus) reduce intestinal toxin exposure and modulate regenerative signaling, whereas prebiotics (e.g., inulin, fructooligosaccharides, resistant starch) promote short-chain fatty acid production and beneficial microbiota shifts that enhance ISC proliferation and differentiation [82].

Importantly, the effects of mycotoxins on ISCs are at least partially reversible, particularly after acute or low-dose exposures [6]. Early or combinatorial interventions—including dietary acids, probiotics/prebiotics, antioxidants, and mycotoxin binders—can restore ISC activity, accelerate epithelial repair, and maintain gut barrier integrity [62]. By directly enhancing ISC function, these strategies mitigate the adverse effects of both acute and chronic mycotoxin exposure, highlighting the central role of stem cell-targeted approaches in preserving intestinal health.

Together, these findings underscore the importance of understanding mycotoxin-specific effects on ISCs, identifying early biomarkers of injury, and developing targeted dietary, microbial, and pharmacological interventions to maintain regenerative capacity and barrier function in both livestock and humans.

## 7. Gaps in Knowledge and Future Directions

Mycotoxins exert multifaceted effects on the intestinal epithelium, encompassing acute injury to epithelial barriers and chronic impairment of regenerative mechanisms. The established paradigm emphasizes their capacity to disrupt tight junctions, induce epithelial apoptosis, and exacerbate inflammatory responses, leading to increased intestinal permeability and heightened disease susceptibility [84,85,86,87,88,89]. Yet, recent advances in ISC biology and organoid technology reveal a deeper layer of vulnerability: mycotoxins directly compromise the stem cell compartment and its supporting niche, thereby hindering the ability of the intestine to recover from injury [89,90,91,92,93,94].

This dual perspective—from barrier damage to stem cell dysfunction—offers a more comprehensive understanding of the intestinal consequences of mycotoxin exposure. It also raises important considerations for risk assessment, since long-term regenerative failure may persist beyond the resolution of acute epithelial injury [95,96,97,98,99]. Moving forward, the integration of advanced in vitro platforms, such as organoid–immune–microbiota co-cultures and organ-on-chip systems, with in vivo models and single-cell approaches will be crucial for uncovering the precise mechanisms by which mycotoxins disrupt epithelial renewal. Such insights will not only refine toxicological evaluation but also inform the development of dietary, microbial, and pharmacological strategies aimed at protecting epithelial resilience in the face of mycotoxin exposure [100,101,102,103,104,105].

Despite significant advances in understanding mycotoxin-induced barrier dysfunction, the impact of these toxins on ISCs and the regenerative potential of the epithelium remains incompletely characterized [106,107,108]. Future research should prioritize direct investigation of ISC vulnerability across different mycotoxin classes, using complementary in vivo models and stem cell-derived organoid systems. Such studies should examine not only cytotoxic effects but also disruptions to key niche signaling pathways, including Wnt/β-catenin, Notch, and EGF, which govern stem cell maintenance, proliferation, and lineage specification [109,110,111,112].

Integration of single-cell transcriptomic and spatial transcriptomic approaches will enable precise mapping of cellular responses within crypts, providing insights into heterogeneous ISC populations and their interactions with neighboring Paneth cells, stromal cells, and immune components. Furthermore, advanced co-culture systems and organ-on-chip platforms that incorporate microbial and immune elements hold promise for elucidating the complex interplay between mycotoxins, the stem cell niche, and gut homeostasis under physiologically relevant conditions [110,111,112,113].

From a translational perspective, there is a critical need to evaluate protective strategies aimed at preserving epithelial regeneration, including targeted nutritional supplementation, microbiota modulation, and pharmacological interventions. Additionally, ISC function and regenerative capacity may represent sensitive endpoints for refining risk assessment, complementing traditional measures of acute cytotoxicity or barrier permeability. By adopting these multidisciplinary approaches, future studies can move beyond descriptive toxicology toward mechanistic understanding, ultimately informing strategies to mitigate long-term intestinal dysfunction caused by mycotoxin exposure.

## Figures and Tables

**Table 1:** Mechanistic effects of mycotoxins on intestinal cell types and epithelial regeneration.

Cell Type	Mycotoxin(s)	Mechanisms/Pathways	Outcome on Epithelial Integrity and Regeneration	References
Enterocytes	DON, ZEA	DON: Inhibition of protein synthesis; endocytosis and degradation of tight junction proteins. ZEA: Modulation of Wnt/β-catenin signaling	Barrier dysfunction, increased susceptibility to microbial translocation	[12,13,15]
Goblet cells	DON, ZEA	PKR- and MAP kinase-dependent repression of resistin-like molecule β (DON); possible modulation of goblet cell differentiation (ZEA)	Compromised mucus barrier, reduced protection against pathogens, decreased epithelial defense	[15,16,17,18]
Paneth cells	DON	Secretion of niche factors supporting ISC survival; mitigation of oxidative stress	Enhanced ISC survival and regenerative potential, mitigation of mycotoxin-induced epithelial injury	[19]
Enteroendocrine cells	DON	Calcium-sensing receptor and TRPA1 channel-mediated release of peptide YY, cholecystokinin, GLP-1	Disrupted satiety signaling, altered gut motility, indirect effects on nutrient absorption, and epithelial health	[20,21]
ISCs	DON, ZEA	DON: IP3R-dependent ER Ca^2+^ signaling; TSC2/mTORC1 pathways downstream of IR and EGFR receptors. ZEA: Dysregulation of Wnt/β-catenin signaling affecting ISC proliferation	Impaired epithelial regeneration, delayed recovery from injury, compromised crypt–villus integrity	[15,19,22,23]

**Table 2:** Effects of Mycotoxins on IELs and cytokine responses.

Mycotoxin	Model	IEL/Lymphocyte Effects	Cytokine/Functional Effects	Reference
DON	Broilers	Reduced CD3+ and CD8+ IELs in duodenal epithelium; altered blood lymphocyte distribution	Not directly measured	[25]
	Mice	Biphasic response: low dose → enhanced Th1 cytokines; high dose → suppressed T-cell proliferation, shifted IEL balance	Increased IL-2, IFN-γ (low dose); suppressed at high dose	[30]
ZEA	Broilers	Reduced CD3+ and CD8+ IELs (with DON, above)	Not directly measured	[25]
	Mice	Reduced T-helper and cytotoxic IEL populations; immune suppression	Altered cytokine expression; estrogenic immune modulation	[31]
T-2 toxin	Pigs	Decreased CD4+ and CD8+ T-cells; reduced B-cell proportions in the ileal wall	Lower IL-2 and IFN-γ secretion	[27]
Fusarium mycotoxin mixtures (DON, ZEA, FB1, T-2)	Chickens	Reduced intestinal lymphocyte proliferation; altered IEL subset distribution; reduced IEL density with coccidial challenge	Impaired local immune surveillance	[26,32]
	Multiple	Reduced IEL-mediated barrier defense; increased susceptibility to pathogens	Synergistic immunosuppression with infections	[33]
Ochratoxin A (OTA)	Chickens	Apoptosis of IELs; reduced T- and B-cell populations in mucosa-associated lymphoid tissues	Impaired mucosal defense	[28]
Aflatoxin B1 (AFB1)	Broilers	Reduced CD3+, CD4+, and CD8+ T-cells in intestinal mucosa	Downregulated IL-2, IFN-γ, IL-10 mRNA	[29]

**Table 3:** Effects of mycotoxins on intestinal barrier function.

Mycotoxin	Cell Type/Animal Model	Targeted TJ/Epithelial Cells	Mechanism of Disruption	Reference
DON	IPEC-J2 cells, Caco-2 cells, weaned piglets	Claudins, Occludin, ZO-1, goblet cells, Paneth cells	Inhibits protein synthesis; induces endocytosis/degradation of TJ proteins via MAPK; mitochondrial dysfunction; PGC1α hijacking; represses mucins/TFFs; IP3R-dependent Ca^2+^ signaling	[12,13,16,19,22,23,35,46]
ZEA	Rats, IPEC-J2 cells	TJ proteins, goblet cells	Modulates Wnt/β-catenin; oxidative stress; alters epithelial homeostasis	[15]
T-2/HT-2 toxins	Porcine small intestinal epithelial cells	TJ proteins	Direct cytotoxicity; oxidative stress; inflammation	[36]
AFB1	Pigs, mice, IPEC-J2 cells	Claudins, Occludin, intestinal epithelium	Reduces TJ gene expression; induces apoptosis; FXR-mediated MLCK signaling	[37,49]
AFM1	IPEC-J2 cells	TJ protein-encoding genes	Decreases TJ gene expression; compromises epithelial integrity	[38]
OTA	Caco-2 cells	Claudins (specific isoforms)	Selective removal of claudin isoforms; increases permeability	[39]

**Table 4:** Nutritional mitigation of mycotoxin effects on ISCs.

Nutritional Intervention	Mycotoxin	Mechanisms/Pathways	Outcome on ISCs and Intestinal Regeneration	References
Recombinant porcine R-Spondin 1	DON	Potentiates Wnt/β-catenin signaling	Expands ISC population along crypt–villus axis; restores proliferation and crypt architecture	[53]
Butyrate	DON	Enhances ISC survival; supports barrier function	Reduces epithelial barrier dysfunction; indirectly promotes ISC-mediated regeneration	[54]
Hydrolyzed Wheat Gluten	DON	Upregulates Wnt/β-catenin signaling	Promotes ISC proliferation and differentiation; alleviates intestinal injury	[59]
Zinc L-Aspartate	DON	Activates Wnt/β-catenin signaling	Enhances ISC activity; protects mucosal integrity	[58]
Lauric Acid	DON	Potentiates Akt/mTORC1/S6K1 signaling	Mitigates ISC damage; supports proliferation and epithelial regeneration	[62]
Methionine & Hydroxyl Analogues	DON	Reactivates Wnt/β-catenin signaling	Improves ISC activity; facilitates repair of DON-induced injury	[57]
L-Carnosine	DON	Regulates Keap1/Nrf2 signaling; reduces oxidative stress	Protects ISCs from ROS-mediated damage; maintains stem cell function	[63]

**Table 6:** Typical experimental and physiological exposure levels of major mycotoxins, species sensitivity, and relevance to human exposure.

Mycotoxin	In Vitro Concentrations (µM)	In Vivo Doses (mg/kg Diet or mg/kg b.w.)	Sensitive Species/Relative Tolerance	Approximate Human Exposure	Regulatory Benchmarks	References
DON	0.1–10 µM (barrier and ISC injury ≥ 1 µM)	0.5–5 mg/kg diet; LOAEL ≈ 0.1 mg/kg b.w./day	Pigs ≈ Humans > Poultry > Ruminants (rumen detoxification)	0.01–0.1 µg/kg b.w./day (dietary intake)	TDI: 1 µg/kg b.w./day (EFSA, 2022); feed limit: ≤0.9 mg/kg (EU)	[5,6,7,12,22,35,51]
ZEA	1–50 µM (estrogenic and epithelial effects ≥ 10 µM)	0.1–5 mg/kg diet; LOAEL ≈ 0.1 mg/kg b.w./day	Pigs (highly sensitive) > Poultry > Ruminants	0.01–0.05 µg/kg b.w./day	TDI: 0.25 µg/kg b.w./day (EFSA); feed limit: ≤0.1 mg/kg (EU)	[15,25,31,36]
AFB1	0.1–5 µM (apoptosis, TJ loss ≥ 1 µM)	0.05–1 mg/kg diet; LOAEL ≈ 0.03 mg/kg b.w./day	Poultry ≈ Pigs > Humans (due to dietary detoxification)	<0.001 µg/kg b.w./day	No safe threshold; limit: ≤2 µg/kg food (EU, FDA)	[29,37,48]
OTA	0.5–10 µM (permeability, ROS ≥ 2 µM)	0.1–2 mg/kg diet; chronic LOAEL ≈ 0.05 mg/kg b.w./day	Pigs > Poultry > Ruminants	0.001–0.01 µg/kg b.w./day	TDI: 0.02 µg/kg b.w./day (EFSA); limit: ≤5 µg/kg (EU)	[28,39,66]
T-2	0.01–1 µM (nanomolar cytotoxicity)	0.1–0.5 mg/kg diet; LOAEL ≈ 0.05 mg/kg b.w./day	Poultry > Pigs > Ruminants	<0.001 µg/kg b.w./day	Indicative TDI: 0.02 µg/kg b.w./day (EFSA)	[27,36,65]

## Data Availability

No new data were created or analyzed in this study.
